# Some Classical and Quantum Aspects of Gravitoelectromagnetism

**DOI:** 10.3390/e22101089

**Published:** 2020-09-27

**Authors:** Giorgio Papini

**Affiliations:** Department of Physics and Prairie Particle Physics Institute, University of Regina, Regina, SK S4S 0A2, Canada; papini@uregina.ca

**Keywords:** gravitoelectromagnetism, condensation phenomena, dark matter

## Abstract

It has been shown that, even in linear gravitation, the curvature of space-time can induce ground state degeneracy in quantum systems, break the continuum symmetry of the vacuum and give rise to condensation in a system of identical particles. Condensation takes the form of a temperature-dependent correlation over distances, of momenta oscillations about an average momentum, of vortical structures and of a positive gravitational susceptibility. In the interaction with quantum matter and below a certain range, gravity is carried by an antisymmetric, second order tensor that satisfies Maxwell-type equations. Some classical and quantum aspects of this type of “gravitoelectromagnetism” were investigated. Gravitational analogues of the laws of Curie and Bloch were found for a one-dimensional model. A critical temperature for a change in phase from unbound to isolated vortices can be calculated using an XY-model.

## 1. Introduction

It is known that, in certain approximations, Einstein equations can be written in the form of Maxwell equations. In linearized gravity, for instance, the Riemann tensor is

(1) $$R_{\mu \nu \sigma \tau }=\frac{1}{2}\left(\varphi_{\mu \sigma ,\nu \tau }+\varphi_{\nu \tau ,\mu \sigma }-\varphi_{\mu \tau ,\nu \sigma }-\varphi_{\nu \sigma ,\mu \tau }\right)\;,$$

which, except for the presence of second order derivatives instead of those of first order, bears a similarity to the electromagnetic field tensor

(2) $$F_{\mu \nu }=\varphi_{\nu ,\mu }-\varphi_{\mu ,\nu }\;.$$

The algebraic and differential identities that these tensors satisfy are also similar. For the electromagnetic field we have

(3) $$F_{\mu \nu }=-F_{\nu \mu }\;;\;\;\;F_{\mu \nu ,\sigma }+F_{\nu \sigma ,\mu }+F_{\sigma \mu ,\nu }=0$$

while for the Riemann tensor we find

(4) $$R_{\mu \nu \sigma \tau }=R_{\nu \mu \sigma \tau }=-R_{\mu \nu \tau \sigma }=R_{\sigma \tau \mu \nu }\;;\;\;R_{\mu \nu \sigma \tau }+R_{\nu \sigma \mu \tau }+R_{\sigma \mu \nu \tau }=0\;;\;\;R_{\mu \nu \sigma \tau ,\rho }+R_{\mu \nu \tau ,\rho }+R_{\mu \nu \rho \sigma ,\tau }=0\;.$$

The last equation of (4) goes under the name of Bianchi identities. The tensors $F_{\mu \nu }$ and $R_{\mu \nu \sigma \tau }$ also appear in the field equations and characterize the fields completely [1]. By separating the “electric” and “magnetic” components according to

(5) $$E_{i}=F_{i0}\;;\;\;\;\;H_{i}=\frac{1}{2}\epsilon_{ijk}F_{jk}\;$$

and

(6) $$E_{ij}=R_{i0j0}\;;\;\;\;\;H_{ij}\frac{1}{2}\epsilon_{ikl}R_{klj0}\;$$

and using (3) and (4), we can write the field equations

(7) $$F_{\mu \nu }{,}^{\nu }=0\;;\;\;\;R_{\mu \nu }-1/2\eta_{\mu \nu }R=0\;,$$

in the form of Maxwell equations [1]

(8) $$\nabla \cdot E=0\;;\;\;\;\nabla \times E+\dot{H}=0\;;\nabla \cdot H=0\;;\;\;\;\nabla \times H-\dot{E}=0\;.$$

In addition, the wave equations $\partial^{2}F_{\mu \nu }=0$ and $\partial^{2}R_{\mu \nu \sigma \tau }=0$ are also satisfied.

The formal similarities just outlined refer to a vacuum. Other similarities and versions of “gravitoelectromagnetism” (GEM) do, however, appear when the interaction of, say, a scalar particle with gravity is considered. In the minimal coupling approximation, a scalar particle of mass *m* obeys the covariant Klein–Gordon equation

(9) $$\left(\nabla_{\mu }\nabla^{\mu }+m^{2}\right)\phi \left(x\right)=0\;,$$

which, in the weak field approximation $g_{\mu \nu }≃\eta_{\mu \nu }+\gamma_{\mu \nu }$ where $\eta_{\mu \nu }$ is the Minkowski metric, and on applying the Lanczos–DeDonder condition

(10) $$\gamma_{\alpha \nu ,}^{\;\;\;\;\;\;\;\nu }-\frac{1}{2}\gamma_{\sigma ,\alpha }^{\sigma }=\Gamma_{\alpha \mu }^{\mu }=0\;,$$

becomes $O(\gamma_{\mu \nu })$

(11) $$\left(\nabla_{\mu }\nabla^{\mu }+m^{2}\right)\phi \left(x\right)≃\left[\eta_{\mu \nu }\partial^{\mu }\partial^{\nu }+m^{2}+\gamma_{\mu \nu }\partial^{\mu }\partial^{\nu }\right]\phi \left(x\right)=0\;.$$

Units $\hbar =c=k_{B}=1$ are used and the notation is as in [2]. In particular, partial derivatives with respect to a variable $x_{\mu }$ are interchangeably indicated by $\partial_{\mu }$, or by a comma followed by μ. $\nabla_{\mu }$ is a covariant derivative. Indices are lowered and raised by means of the metric tensor $g_{\mu \mu }=\eta_{\mu \nu }+\gamma_{\mu \nu }$, $g^{\mu \nu }=\eta^{\mu \nu }-\gamma^{\mu \nu }$ and gravitational contributions are kept only up to $O(\gamma_{\mu \nu })$.

Equation (11) has the exact solution [3,4,5]

(12) $$\phi \left(x\right)=\left(1-i\Phi_{G}\left(x\right)\right)\phi_{0}\left(x\right)\;,$$

where $\phi_{0}\left(x\right)$ is a plane wave solution of the free Klein–Gordon equation,

(13) $$\Phi_{G}\left(x\right)=-\frac{1}{2}\int_{P}^{x}dz^{\lambda }\left(\gamma_{\alpha \lambda ,\beta }\left(z\right)-\gamma_{\beta \lambda ,\alpha }\left(z\right)\right)\left(x^{\alpha }-z^{\alpha }\right)k^{\beta }$$

$$+\frac{1}{2}\int_{P}^{x}dz^{\lambda }\gamma_{\alpha \lambda }\left(z\right)k^{\alpha }=\int_{P}^{x}dz^{\lambda }K_{\lambda }\left(z,x\right)\;,$$

*P* is some fixed base event that may be omitted for simplicity in what follows and

(14) $$K_{\lambda }\left(z,x\right)=-\frac{1}{2}\left[\left(\gamma_{\alpha \lambda ,\beta }\left(z\right)-\gamma_{\beta \lambda ,\alpha }\left(z\right)\right)\left(x^{\alpha }-z^{\alpha }\right)-\gamma_{\beta \lambda }\left(z\right)\right]k^{\beta }\;.$$

Two-point functions such as (14) belong to the family of world-functions introduced into Riemannian geometry by Ruse [6] and Synge [7] and are used in general relativity to study the curvature structure of space-time. Here they also introduce elements of the topology of space-time, as can be seen by taking the derivatives with respect to *z* of the function $\Phi_{g}$ defined by

(15) $$\frac{\partial \Phi_{g}\left(z\right)}{\partial z^{\sigma }}=-\frac{1}{2}\left[\left(\gamma_{\alpha \sigma ,\beta }\left(z\right)-\gamma_{\beta \sigma ,\alpha }\left(z\right)\right)\left(x^{\alpha }-z^{\alpha }\right)-\gamma_{\beta \sigma }\left(z\right)\right]k^{\beta }\;,$$

which coincides with (14). One obtains

(16) $$\frac{\partial^{2}\Phi_{g}\left(z\right)}{\partial z^{\tau }\partial z^{\sigma }}-\frac{\partial^{2}\Phi_{g}\left(z\right)}{\partial z^{\sigma }\partial z^{\tau }}=R_{\alpha \beta \sigma \tau }\left(x^{\alpha }-z^{\alpha }\right)k^{\beta }\equiv \left[\partial z_{\tau },\partial z_{\sigma }\right]\Phi_{g}\left(z\right)=K_{\sigma ,\tau }\left(z,x\right)-K_{\tau ,\sigma }\left(z,x\right)={\tilde{F}}_{\tau \sigma }\left(z\right)\;,$$

where $R_{\alpha \beta \sigma \tau }$ is the linearized Riemann tensor (1). It follows from (16) that $\Phi_{g}$ is not single-valued, and that after a gauge transformation, $K_{\alpha }$ satisfies the equations

(17) $$\partial z_{\alpha }K^{\alpha }=\partial_{\alpha }K^{\alpha }=\frac{\partial^{2}\Phi_{g}}{\partial z_{\sigma }\partial z^{\sigma }}=0$$

that

(18) $$\partial_{z}^{2}K_{\lambda }=-\frac{k^{\beta }}{2}\left[\left(\partial^{2}\left(\gamma_{\alpha \lambda ,\beta }\right)-\partial^{2}\left(\gamma_{\beta \lambda ,\alpha }\right)\right)\left(x^{\alpha }-z^{\alpha }\right)+\partial^{2}\gamma_{\beta \lambda }\right]\equiv j_{\lambda }$$

identically, and that $j_{\lambda }{,}^{\lambda }=0$ because of (17), while the equation

(19) $$\left[\partial z_{\mu },\partial z_{\nu }\right]\partial z_{\alpha }\Phi_{g}=-\left({\tilde{F}}_{\mu \nu ,\alpha }+{\tilde{F}}_{\alpha \mu ,\nu }+{\tilde{F}}_{\mu \alpha ,\nu }\right)=0\;,$$

holds everywhere. Therefore, $K_{\alpha }$ is regular everywhere, but $\Phi_{g}$ is singular because the double derivatives of $\Phi_{g}$ do not commute. From (16) one gets $\partial_{z}^{2}\partial_{\mu }\Phi_{g}=j_{\mu }$ that can be solved to yield $\partial_{\mu }\Phi_{g}$. Notice also that

(20) $$\partial^{\sigma }{\tilde{F}}_{\tau \sigma }=-j_{\tau }\;$$

and that again $j_{\tau }{,}^{\tau }=0$. Equations (19) and (20) are Maxwell-type equations, and (18) is similar to the equation satisfied by the vector potential in electromagnetism. In essence, the scalar particle of mass *m* “sees” a gravitational field that, under the circumstances discussed in Section 6, is vectorial, rather than tensorial, and acquires a generalized momentum that generates the correct geometrical optics and index of refraction, as shown below. The new quantities $K_{\lambda },{\tilde{F}}_{\alpha \beta },P_{\alpha }$ contain the original particle momentum $k_{\beta }$. They therefore acquire, upon quantization, the characteristics of “quasiparticles”. This alternate procedure also brings to the fore the elusive “electromagnetic” character of gravity and the “electromagnetic” properties so acquired persist at higher order in $\gamma_{\mu \nu }$. In fact, to any order *n* of $\gamma_{\mu \nu }$, the solution of (9) can be written as

(21) $$\phi \left(x\right)=\Sigma_{n}\phi_{\left(n\right)}\left(x\right)=\Sigma_{n}e^{-i{\hat{\Phi }}_{G}}\phi_{\left(n-1\right)}\;.$$

where ${\hat{\Phi }}_{G}$ is the operator obtained from (13) by replacing $k^{\alpha }$ with $i\partial^{\alpha }$. In what follows, the approximations are always carried out to $O(\gamma_{\mu \nu })$ for simplicity.

The fundamental reason for the appearance of gravitational quasiparticles resides in (12) and (13) and has a geometrical explanation. Equations (12) and (13) represent a space-time transformation of the vacuum that makes the ground state degenerate, breaks the continuous symmetry of the system and leads to the phenomenon of condensation. This follows from the well-known fact that the original, rotation-invariant system $\phi_{0}$, transported along a closed path Γ bounding a small surface $df^{\beta \delta }$, changes, because of the curvature of space-time, by

(22) $$\delta \phi =\phi -\phi_{0}≃\frac{i}{4}\int df^{\beta \delta }R_{\mu \nu \delta \beta }\left[J^{\mu \nu },\phi_{0}\right]\;,$$

where $J^{\mu \nu }$ is the angular momentum operator and

(23) $$\left[J^{\mu \nu },\phi_{0}\right]=i\left\{\left(x^{\mu }-z^{\mu }\right)\frac{\partial \phi_{0}}{\partial x^{\nu }}-\left(x^{\nu }-z^{\nu }\right)\frac{\partial \phi_{0}}{\partial x^{\mu }}\right\}\;.$$

Equation (22) can be obtained by applying Stokes’ theorem to (12) and (13). The rotational invariant system $\phi_{0}$ therefore acquires a privileged direction (that of rotation), on being translated along the closed path Γ in curved space-time. The direction of rotation due to $J_{\mu \nu }$ breaks the original symmetry. The new space-time-dependent ground state (12) reflects the lack of symmetry under rotation. Thus the topological singularity that appears in $\Phi_{g}$ is supplied by the geometry of space-time itself.

It is possible to construct more complex solutions of (11), such as a closed rectangular loop, or ring, by combining two vortex-type solutions (12) and (13) with opposite directions, and in general, by linear superposition of the solution found. In short, the curvature of space-time breaks the original symmetry by introducing preferred directions.

There are consequences for the scalar particle and the gravitational field. Notwithstanding the use of a quantum wave equation for ϕ, the fields $\gamma_{\mu \nu },K_{\lambda }$ and ${\tilde{F}}_{\alpha \beta }$ are classical. The transformation (12) and (13) does not affect only the field $\phi_{0}$, but the other fields as well, as shown in Section 2, Section 3, Section 4 and Section 5 where dispersion relations, geometrical optics, particle motion and the appearance of quasiparticles are discussed. Initially the fields are represented by $\gamma_{\mu \nu }$ which is classical, and by the field $\phi_{0}$, which satisfies a quantum equation and has rotational symmetry. Finally, the gravitational field is represented by $K_{\lambda }$ which is classical and contains a topological singularity generated by the curvature of space-time, while ϕ, which satisfies a quantum equation, is no longer rotational invariant. This transformation is discussed at length in the following sections together with various aspects of the interaction of $K_{\lambda }$ with matter. In Section 6 we introduce a simple model Lagrangian that illustrates how condensation affects the quantum system considered. Section 7 and Section 8 are concerned with statistical aspects of an ensemble of scalar particles in a gravitational background and the origin of a correlation length and alignment per particle, together with analogues of Curie and Bloch laws and spin waves in the ensemble. A summary and conclusions are contained in Section 9.

Solutions similar to (12) and (13) have also been found for particles with spin-2 [8], spin-1 [9] and spin-1/2 [3]. We refer to the original papers for their derivations and discussions regarding minimal coupling terms. The common feature of the solutions given in [3,4,5,6,7,8,10] is the presence of the term (23) due to curved space-time.

We report, in particular, the results for spin-2 particles in the following section when discussing geometrical optics.

## 2. Dispersion Relations, Geometrical Optics and Particle Motion

By using Schroedinger’s logarithmic transformation [11] $\phi =e^{-iS}$ we can pass from the KG Equation (9) to the quantum Hamilton–Jacobi equation. We find to first order in $\gamma_{\mu \nu }$

(24) $$i\left(\eta^{\mu \nu }-\gamma^{\mu \nu }\right)\partial_{\mu }\partial_{\nu }S-\left(\eta^{\mu \nu }-\gamma^{\mu \nu }\right)\partial_{\mu }S\partial_{\nu }S+m^{2}=0\;,$$

where

(25) $$S=k^{\beta }\left\{x_{\beta }+\frac{1}{2}\int^{x}dz^{\lambda }\gamma_{\beta \lambda }\left(z\right)-\frac{1}{2}\int^{x}dz^{\lambda }\left(\gamma_{\alpha \lambda ,\beta }\left(z\right)-\gamma_{\beta \lambda ,\alpha }\left(z\right)\right)\left(x^{\alpha }-z^{\alpha }\right)\right\}$$

$$\equiv k_{\alpha }x^{\alpha }+A+B\;.$$

It is well-known that the Hamilton–Jacobi equation is equivalent to Fresnel’s wave equation in the limit of large frequencies [11]. However, at smaller or moderate frequencies the complete Equation (24) should be used. We follow this path. By substituting (12) into the first term of (24), we obtain

(26) $$i\left(\eta^{\mu \nu }-\gamma^{\mu \nu }\right)\partial_{\mu }\partial_{\nu }S=i\eta^{\mu \nu }\partial_{\mu }\left(k_{\nu }+\Phi_{G,\nu }\right)-i\gamma^{\mu \nu }\partial_{\mu }k_{\nu }=i\eta^{\mu \nu }\Phi_{G,\mu \nu }=ik_{\alpha }\eta^{\mu \nu }\Gamma_{\mu \nu }^{\alpha }=0\;,$$

on account of (10). This part of (24) is usually neglected in the limit ℏ→0. Here it vanishes as a consequence of solution (12). The remaining terms of (24) yield the classical Hamilton–Jacobi equation

(27) $$\left(\eta^{\mu \nu }-\gamma^{\mu \nu }\right)\partial_{\mu }S\partial_{\nu }S-m^{2}=\gamma^{\mu \nu }k_{\mu }k_{\nu }-2k^{\mu }\Phi_{G,\mu }=0\;,$$

because $k^{\mu }\Phi_{G,\mu }=1/2\gamma^{\mu \nu }k_{\mu }k_{\nu }$. Equation (12) is therefore a solution of the more general quantum Equation (24). It also follows that the particle acquires the generalized “momentum”

(28) $$P_{\mu }=k_{\mu }+\Phi_{G,\mu }=k_{\mu }+\frac{1}{2}\gamma_{\alpha \mu }k^{\alpha }-\frac{1}{2}\int^{x}dz^{\lambda }\left(\gamma_{\mu \lambda ,\beta }\left(z\right)-\gamma_{\beta \lambda ,\mu }\left(z\right)\right)k^{\beta }\;,$$

that satisfies the dispersion relation

(29) $$P_{\mu }P^{\mu }\equiv m_{e}^{2}=m^{2}\left(1+\gamma_{\alpha \mu }\left(x\right)u^{\alpha }u^{\mu }-\frac{1}{2}\int^{x}dz^{\lambda }\left(\gamma_{\mu \lambda ,\beta }\left(z\right)-\gamma_{\beta \lambda ,\mu }\left(z\right)\right)u^{\mu }u^{\beta }\right)\;.$$

The integral in (29) vanishes because $u^{\mu }u^{\beta }$ is contracted on the antisymmetric tensor in round brackets. The effective mass $m_{e}$ is not in general constant. In this connection too we can speak of quasiparticles. The medium in which the scalar particles propagate is here represented by space-time.

By differentiating with respect to $x_{\mu }$

(30) $$\Phi_{G,\mu }=K_{\mu }\left(x,x\right)+\int^{x}dz^{\lambda }\partial_{\mu }K_{\lambda }\left(z,x\right)\;,$$

and

(31) $$\Phi_{G,\mu \nu }=K_{\mu ,\nu }\left(x,x\right)+\partial_{\nu }\int^{x}dz^{\lambda }\partial_{\mu }K_{\lambda }\left(z,x\right)=k_{\alpha }\Gamma_{\mu \nu }^{\alpha }\;,$$

where $\Gamma_{\mu \nu }^{\alpha }$ are the Christoffel symbols, and by differentiating (28) we obtain the covariant derivative of $P_{\mu }$

(32) $$\frac{DP_{\mu }}{Ds}=m\left[\frac{du_{\mu }}{ds}+\frac{1}{2}\left(\gamma_{\alpha \mu ,\nu }-\gamma_{\mu \nu ,\alpha }+\gamma_{\alpha \nu ,\mu }\right)u^{\alpha }u^{\nu }\right]$$

$$=m\left(\frac{du_{\mu }}{ds}+\Gamma_{\alpha ,\mu \nu }u^{\alpha }u^{\nu }\right)=\frac{Dk^{\mu }}{Ds}\;.$$

This result is independent of any choice of field equations for $\gamma_{\mu \nu }$. We see from (32) that, if $k_{\mu }$ follows a geodesic, then $\frac{DP_{\mu }}{Ds}=0$ and $\frac{Dm_{e}^{2}}{Ds}=0$. The classical equations of motion are therefore contained in (32), but it would require the particle described by (9) to just choose a geodesic, among all the paths allowed to a quantum particle.

We also obtain, from (24), $\sqrt{{\left(\partial_{i}S\right)}^{2}}=\pm \sqrt{-m^{2}+{\left(\partial_{0}S\right)}^{2}-\gamma^{\mu \nu }\partial_{\mu }S\partial_{\nu }S}$, which, in the absence of gravity, gives $k_{i}^{2}=\sqrt{-m^{2}+k_{0}^{2}}$, as expected. Remarkably, (28) is an exact integral of (32) which can itself be integrated exactly to give the particle’s motion

(33) $$X_{\mu }=x_{\mu }+\frac{1}{2}\int^{x}dz^{\lambda }\left\{\gamma_{\mu \lambda }-\left(\gamma_{\alpha \lambda ,\mu }-\gamma_{\mu \lambda ,\alpha }\right)\left(x^{\alpha }-z^{\alpha }\right)\right\}\;.$$

Higher order approximations to the solution of (9) can be obtained by using (21), which plays a dynamical role akin to Feynman’s path integral formula [10]. In (21), however, it is the solution itself that is varied by successive approximations, rather than the particle’s path.

In order to gain some insight into the formalism, we derive the geometrical optics approximation for a spin-2 particle. The covariant wave equation for spin-2 particles is

(34) $$\nabla_{\alpha }\nabla^{\alpha }\Phi_{\mu \nu }+m^{2}\Phi_{\mu \nu }=0,$$

The first order in $\gamma_{\mu \nu }$, (34) can be written in the form

(35) $$\left(\eta^{\alpha \beta }-\gamma^{\alpha \beta }\right)\partial_{\alpha }\partial_{\beta }\Phi_{\mu \nu }+R_{\sigma \mu }\Phi_{\nu }^{\sigma }+R_{\sigma \nu }\Phi_{\mu }^{\sigma }-2\Gamma_{\mu \alpha }^{\sigma }\partial^{\alpha }\Phi_{\nu \sigma }-2\Gamma_{\nu \alpha }^{\sigma }\partial^{\alpha }\Phi_{\mu \sigma }+m^{2}\Phi_{\mu \nu }=0,$$

where $R_{\mu \beta }=-\left(1/2\right)\partial_{\alpha }\partial^{\alpha }\gamma_{\mu \beta }$ is the linearized Ricci tensor of the background metric and $\Gamma_{\sigma \mu ,\alpha }=1/2\left(\gamma_{\alpha \sigma ,\mu }+\gamma_{\alpha \mu ,\sigma }-\gamma_{\sigma \mu ,\alpha }\right)$ is the corresponding Christoffel symbol of the first kind. The solution is

(36) $$\begin{array}{c} \Phi_{\mu \nu }\left(x\right)=\phi_{\mu \nu }\left(x\right)+\frac{1}{2}\int_{P}^{x}dz^{\lambda }\gamma_{\alpha \lambda }\left(z\right)\partial^{\alpha }\phi_{\mu \nu }\left(x\right)-\frac{1}{2}\int_{P}^{x}dz^{\lambda }\left(\gamma_{\alpha \lambda ,\beta }\left(z\right)-\gamma_{\beta \lambda ,\alpha }\left(z\right)\right)\left[\left(x^{\alpha }-z^{\alpha }\right)\partial^{\beta }+iS^{\alpha \beta }\right]\phi_{\mu \nu }\left(x\right)\\ -\frac{i}{2}\int_{P}^{x}dz^{\lambda }\gamma_{\beta \sigma ,\lambda }\left(z\right)T^{\beta \sigma }\phi_{\mu \nu }\left(x\right)\;, \end{array}$$

where

(37) $$\begin{array}{ccc} S^{\alpha \beta }\phi_{\mu \nu } & \equiv & \frac{i}{2}\left(\delta_{\sigma }^{\alpha }\delta_{\mu }^{\beta }\delta_{\nu }^{\tau }-\delta_{\sigma }^{\beta }\delta_{\mu }^{\alpha }\delta_{\nu }^{\tau }+\delta_{\sigma }^{\alpha }\delta_{\nu }^{\beta }\delta_{\mu }^{\tau }-\delta_{\sigma }^{\beta }\delta_{\nu }^{\alpha }\delta_{\mu }^{\tau }\right)\phi_{\tau }^{\sigma }\\ T^{\beta \sigma }\phi_{\mu \nu } & \equiv & i\left(\delta_{\mu }^{\beta }\delta_{\nu }^{\tau }+\delta_{\nu }^{\beta }\delta_{\mu }^{\tau }\right)\phi_{\tau }^{\sigma }\;. \end{array}$$

From $S^{\alpha \beta }$ one constructs the rotation matrices $S_{i}=-2i\epsilon_{ijk}S^{jk}$ that satisfy the commutation relations $\left[S_{i},S_{j}\right]=i\epsilon_{ijk}S_{k}$. The spin-gravity interaction is therefore contained in the term

(38) $$\Phi_{\mu \nu }^{\prime }\equiv -\frac{i}{2}\int_{P}^{x}dz^{\lambda }\left(\gamma_{\alpha \lambda ,\beta }-\gamma_{\beta \lambda ,\alpha }\right)S^{\alpha \beta }\phi_{\mu \nu }\left(x\right)=\frac{1}{2}\int_{P}^{x}dz^{\lambda }\left[\left(\gamma_{\sigma \lambda ,\mu }-\gamma_{\mu \lambda ,\sigma }\right)\phi_{\nu }^{\sigma }+\left(\gamma_{\sigma \lambda ,\nu }-\gamma_{\nu \lambda ,\sigma }\right)\phi_{\mu }^{\sigma }\right].$$

The solution (36) is invariant under the gauge transformations $\gamma_{\mu \nu }\to \gamma_{\mu \nu }-\xi_{\mu ,\nu }-\xi_{\nu ,\mu }$, where $\xi_{\mu }$ are small quantities of the first order. If, in fact, we choose a closed integration path Γ, Stokes’ theorem transforms the first three integrals of (36) into $1/4\int_{\Sigma }d\sigma^{\lambda \kappa }R_{\lambda \kappa \alpha \beta }\left(L^{\alpha \beta }+S^{\alpha \beta }\right)\phi_{\mu \nu }$, where Σ is the surface bound by Γ, $J^{\alpha \beta }=L^{\alpha \beta }+S^{\alpha \beta }$ is the total angular momentum of the particle and $R_{\lambda \kappa \alpha \beta }$ is the linearized Riemann tensor (1). For the same path Γ the integral involving $T^{\beta \sigma }$ in (36) behaves like a gauge term and may therefore be dropped. For the same closed paths, (36) gives

(39) $$\Phi_{\mu \nu }≃\left(1-i\xi \right)\phi_{\mu \nu }=\left(1-\frac{i}{4}\int_{\Sigma }d\sigma^{\lambda \kappa }R_{\lambda \kappa \alpha \beta }J^{\alpha \beta }\right)\phi_{\mu \nu }\;,$$

which obviously is covariant and gauge invariant, and where $\phi_{\mu \nu }$ satisfies the field-free equation.

Neglecting spin effects [12,13], we get from (36)

(40) $$\Phi_{\mu \nu }^{0}=\phi_{\mu \nu }\left(x\right)+\frac{1}{2}\int_{P}^{x}dz^{\lambda }\gamma_{\alpha \lambda }\left(z\right)\partial^{\alpha }\phi_{\mu \nu }\left(x\right)-\frac{1}{2}\int_{P}^{x}dz^{\lambda }\left(\gamma_{\alpha \lambda ,\beta }\left(z\right)-\gamma_{\beta \lambda ,\alpha }\left(z\right)\right)\left[\left(x^{\alpha }-z^{\alpha }\right)\partial^{\beta }\right]\phi_{\mu \nu }\left(x\right).$$

The general relativistic deflection of a spin-2 particle in a gravitational field follows immediately from $\Phi_{\mu \nu }^{0}$. By assuming, for simplicity, that the spin-2 particles are massless and propagate along the *z*-direction, so that $k^{\alpha }≃\left(k,0,0,k\right)$, and $ds^{2}=0$ or dt=dz, using plane waves for $\phi_{\mu \nu }$ and writing

(41) $$\chi =k_{\sigma }x^{\sigma }-\frac{1}{4}\int_{P}^{x}dz^{\lambda }\left(\gamma_{\alpha \lambda ,\beta }\left(z\right)-\gamma_{\beta \lambda ,\alpha }\left(z\right)\right)\left[\left(x^{\alpha }-z^{\alpha }\right)k^{\beta }-\left(x^{\beta }-z^{\beta }\right)k^{\alpha }\right]+\frac{1}{2}\int_{P}^{x}dz^{\lambda }\gamma_{\alpha \lambda }\left(z\right)k^{\alpha }\;,$$

the particle momentum is

(42) $${\tilde{k}}_{\sigma }=\frac{\partial \chi }{\partial x^{\sigma }}\equiv \chi_{,\sigma }=k_{\sigma }-\frac{1}{2}\int_{P}^{x}dz^{\lambda }\left(\gamma_{\sigma \lambda ,\beta }-\gamma_{\beta \lambda ,\sigma }\right)k^{\beta }+\frac{1}{2}\gamma_{\alpha \sigma }k^{\alpha }\;.$$

It then follows from (42) that χ satisfies the eikonal equation $g^{\alpha \beta }\chi_{,\alpha }\chi_{,\beta }=0$.

The calculation of the deflection angle is particularly simple to do if the background metric is

(43) $$\gamma_{00}=2\varphi \left(r\right)\;\;,\;\gamma_{ij}=2\varphi \left(r\right)\delta_{ij}\;,$$

where φ(r)=−GM/r and $r=\sqrt{x^{2}+y^{2}+z^{2}}$, which is frequently used in gravitational lensing. For this metric, χ is given by

(44) $$\chi ≃-\frac{k}{2}\int_{P}^{x}\left[\left(x-x^{\prime }\right)\phi_{,z^{\prime }}dx^{\prime }+\left(y-y^{\prime }\right)\phi_{,\;z^{\prime }}dy^{\prime }-2\left[\left(x-x^{\prime }\right)\phi_{,\;x^{\prime }}+\left(y-y^{\prime }\right)\phi_{,\;y^{\prime }}\right]dz^{\prime }\right]+k\int_{P}^{x}dz^{\prime }\phi \;.$$

The space components of the momentum are therefore

(45) $$\begin{array}{ccc} {\tilde{k}}_{1} & = & 2k\int_{P}^{x}\left(-\frac{1}{2}\frac{\partial \varphi }{\partial z}\;dx+\frac{\partial \varphi }{\partial x}dz\right)\;, \end{array}$$

(46) $$\begin{array}{ccc} {\tilde{k}}_{2} & = & 2k\int_{P}^{x}\left(-\frac{1}{2}\frac{\partial \varphi }{\partial z}\;dy+\frac{\partial \varphi }{\partial y}dz\right)\;, \end{array}$$

(47) $$\begin{array}{ccc} {\tilde{k}}_{3} & = & k(1+\varphi )\;. \end{array}$$

We find

(48) $$\tilde{k}={\tilde{k}}_{⊥}+k_{3}\;e_{3}\;,\;{\tilde{k}}_{⊥}=k_{1}\;e_{1}+k_{1}\;e_{2}\;,$$

where ${\tilde{k}}_{⊥}$ is the component of the momentum orthogonal to the direction of propagation of the particles.

Since only phase differences are physical, it is convenient to choose the space-time path by placing the particle source at a distance very large relative to the dimensions of *M*, while the generic point is located at *z* along the *z* direction and z≫x,y. Equations (45)–(47) simplify to

(49) $$\begin{array}{ccc} {\tilde{k}}_{1} & = & 2k\int_{-\infty }^{z}\frac{\partial \varphi }{\partial x}dz=k\;\frac{2GM}{R^{2}}\;x\left(1+\frac{z}{r}\right)\;, \end{array}$$

(50) $$\begin{array}{ccc} {\tilde{k}}_{2} & = & 2k\int_{-\infty }^{z}\frac{\partial \varphi }{\partial y}dz=k\;\frac{2GM}{R^{2}}\;y\left(1+\frac{z}{r}\right)\;, \end{array}$$

(51) $$\begin{array}{ccc} {\tilde{k}}_{3} & = & k(1+\varphi )\;, \end{array}$$

where $R=\sqrt{x^{2}+y^{2}}$. By defining the deflection angle as

(52) $$\tan\theta =\frac{{\tilde{k}}_{⊥}}{{\tilde{k}}_{3}}\;,$$

one finds

(53) $$\tan\theta ∼\theta ∼\frac{2GM}{R}\left(1+\frac{z}{r}\right)\;,$$

which, in the limit z→∞, yields the Einsteinian result

(54) $$\theta_{M}∼\frac{4GM}{R}\;.$$

The index of refraction can be derived from the known equation $n=\tilde{k}/{\tilde{k}}_{0}$. By choosing the direction of propagation of the particle along the $x^{3}—$axis, and using (42), one finds

(55) $$n≃1+\frac{1}{k_{0}}\left(\chi_{,3}-\chi_{,0}\right)-\frac{m^{2}}{2k_{0}^{2}}\left(1-\frac{1}{k_{0}}\chi_{,0}\right)$$

and, again, for $k_{0}\gg m$, or for vanishing *m*,

(56) $$n≃1+\frac{1}{2k_{0}}\left[-\int_{P}^{x}dz^{\lambda }\left(\gamma_{3\lambda ,\beta }-\gamma_{\beta \lambda ,3}\right)k^{\beta }+\gamma_{\alpha 3}k^{\alpha }+\int_{P}^{x}dz^{\lambda }\left(\gamma_{0\lambda ,\beta }-\gamma_{\beta \lambda ,0}\right)k^{\beta }-\gamma_{\alpha 0}k^{\alpha }\right]\;.$$

In the case of the metric (43), one gets the result

(57) $$n≃1+\int_{P}^{x}dz^{0}\gamma_{00,3}=1-\frac{2GM}{r}\;.$$

## 3. The Gravitational WKB Problem

We now study the propagation of a scalar field in a gravitational background. We know, from standard quantum mechanics [14], that *S* of (25) develops an imaginary part when the particle tunnels through a potential. This imaginary contribution is interpreted as the transition amplitude across the classically forbidden region, which is therefore given by [15]

(58) $$T=\exp\left[-2Im(S)\right]=exp\left\{-2Im\left[\ln\left(\Sigma_{n}\exp\left(-i{\hat{\Phi }}_{G}\phi_{n-1}\right)\right)\right]\right\}\;.$$

To $O(\gamma_{\mu \nu })$, (58) becomes

(59) $$T=exp\left\{-2Im\left[x_{\beta }+\frac{1}{2}\oint dz^{\lambda }\gamma_{\beta \lambda }\left(z\right)-\frac{1}{2}\oint dz^{\lambda }\left(\gamma_{\alpha \lambda ,\beta }\left(z\right)-\gamma_{\beta \lambda ,\alpha }\left(z\right)\right)\left(x^{\alpha }-z^{\alpha }\right)\right]k^{\beta }\right\}\;,$$

for a space-time path traversing the gravitational background from −∞ to +∞ and back as it must in order to make (59) invariant. Assuming a Boltzmann distribution for the particles $T=e^{-k_{0}/T}$, where *T* is the temperature, we find, in general coordinates,

(60) $$T=k_{0}/Im\left\{2k^{\beta }\left[x_{\beta }+\frac{1}{2}\oint dz^{\lambda }\gamma_{\beta \lambda }\left(z\right)-\frac{1}{2}\oint dz^{\lambda }\left(\gamma_{\alpha \lambda ,\beta }\left(z\right)-\gamma_{\beta \lambda ,\alpha }\right)\left(x^{\alpha }-z^{\alpha }\right)\right]\right\}\;.$$

The intended application here is to the propagation problem in Rindler space given by

(61) $$ds^{2}={\left(1+ax\right)}^{2}{\left(dx^{0}\right)}^{2}-{\left(dx\right)}^{2}\;,$$

with a horizon at x=−1/a, where $a^{2}=a_{\alpha }a^{\alpha }$ is the constant proper acceleration measured in the rest frame of the Rindler observer. We note that, a priori, our approach is ill-suited to treat this problem which is frequently tackled in the literature starting from exact or highly symmetric solutions of the KG equation [16]. In fact, the weak field approximation $|\gamma_{\mu \nu }\left|<\right|\eta_{\mu \nu }|$ may become inadequate close to the horizon, from where the imaginary part of T comes, for some systems of coordinates. This requires attention, as discussed below. Nonetheless, the approximation has interesting features, such as the presence of $k_{\alpha }$ in (60) and manifest covariance and invariance under canonical transformations.

It is convenient, for our purposes, to use the Schwarzschild-like form for (61) using the transformation [17]

(62) $$x^{0}=\frac{1}{a}\sqrt{1+2ax^{\prime }}\sinh\left(at^{\prime }\right)\;,\;\;x^{1}=\frac{1}{a}\sqrt{1+2ax^{\prime }}\cosh\left(at^{\prime }\right)$$

for $x^{1}\geq -1/2a$ and the same transformation with the hyperbolic functions interchanged for $x^{1}\leq -1/2a$. The resulting metric is

(63) $$ds^{2}=\left(1+2ax^{\prime }\right){\left(dt^{\prime }\right)}^{2}-\frac{1}{1+2ax^{\prime }}{\left(dx^{\prime }\right)}^{2}\;,$$

for which the horizon is at $x^{\prime }=-1/2a$. From (63) we find $\gamma_{00}=2ax^{\prime },\gamma_{11}=2ax^{\prime }/\left(1+2ax^{\prime }\right)$. If $\gamma_{00}$ and $\gamma_{11}$ represent corrections to the Minkowski metric, we must have $|\gamma_{00}/g_{00}\left|<1,\right|\gamma_{11}/g_{11}|<1$ for any a>0. The approximation therefore remains valid for $-1/4a<x^{\prime }<1/2a$. This is sufficient for our calculation. We now write the terms *A* and *B*, defined in (25), for the metric (63) explicitly. We find

(64) $$A=\frac{k^{0}}{2}\int dz^{0}\gamma_{00}+\frac{k^{1}}{2}\int dz^{1}\gamma_{11}\;,$$

and, by taking the reference point $x^{\mu }=0$,

(65) $$B=-\frac{k^{0}}{2}\int dz^{0}\gamma_{00,1}z^{1}+\frac{k^{1}}{2}\int dz^{0}\gamma_{00,1}z^{0}\;.$$

The explicit expressions for *A* and *B* confirm the fact that T receives contributions from both time and space parts of *S*, as pointed out in [17]. On the other hand, this is expected of a fully covariant approach.

The first integrals in *A* and *B* cancel each other. The second integral in *A* can be calculated by contour integration by writing $z^{1}=-1/2a+\epsilon e^{i\theta }$. The result $Im\int_{-\infty }^{\infty }dz^{1}z^{1}k^{1}/\left(1+2az^{1}\right)=-k^{1}\pi /4a^{2}$ yields a vanishing contribution because $k^{1}$ reverses its sign on the return trip. The last integral in *B* is real. The term $k_{0}\Delta t^{\prime }$ in (25) contributes the amount $k^{0}\left(-i\pi /2a\right)2$ because for a round trip the horizon is crossed twice and each time $at^{\prime }\to at^{\prime }-i\pi /2$ because of (62). The remaining term of (60) gives $k_{1}\Delta x^{\prime }=k_{1}x^{\prime }-\left(-k_{1}\right)\left(-x^{\prime }\right)=0$. The final result is therefore

(66) $$T=\frac{a}{2\pi }\;,$$

which is independent of $k^{1}$ and coincides with the usual Unruh temperature [18,19]. This result, with the replacement $a\to a/\sqrt{1-a^{2}/A^{2}}$, where A=2m is the maximal acceleration, also confirms a recent calculation [20] regarding particles whose accelerations has an upper limit. Equation (66) comes in fact from the term $k_{0}\Delta t^{\prime }$ that does not contain derivatives of $\gamma_{\mu \nu }$. The difference from [20] and from [17], is, however, represented by the form of (59) of the decay rate [15], which carries a factor 2 in the exponential, as required by our invariant approach.

Despite its limitations, the approximation already reproduces (66) at $O(\gamma_{\mu \nu })$. Additional terms of (58) are expected to contain corrections to (66). We note, however, that for a closed space-time path the last integral in (59) and (60) becomes $\int_{\Sigma }d\sigma^{\mu \lambda }R_{\mu \lambda \alpha \beta }J^{\alpha \beta }$, where Σ is the surface bounded by the path [3], and has an imaginary part if $R_{\mu \nu \alpha \beta }$ has singularities. This eventuality may call for a complete quantum theory of gravity [21].

## 4. “Gravitomagnetic” and “Gravitoelectric” Charges. $K_{\lambda }$ in Interaction

*i. Charge densities.* From (20) and (6) we obtain $div\tilde{H}=\left[-R_{\alpha \beta 32,1}+R_{\alpha \beta 31,2}-R_{\alpha \beta 21,3}\right]\left(x^{\alpha }-z^{\alpha }\right)k^{\beta }+\left(-R_{1\beta 32}+R_{2\beta 31}-R_{3\beta 21}\right)k^{\beta }=0$ on account of (4). There are therefore no “magnetic charges” in this version of GEM.

From (18), the definition of $J_{\alpha \beta }$ in (23) and the Einstein equations $\partial^{2}\gamma^{\beta \alpha }=R^{\beta \alpha }=\left(8\pi G/c^{4}\right)T^{\beta \alpha }$, we find $j^{\tau }=\left(8\pi G/c^{4}\right)\left(T^{\beta \tau }k_{\beta }\right)$, which, integrated over all space, is the work done by the field to displace the particle by $x_{\alpha }-z_{\alpha }$ in a time dt. The charge density is $j^{0}$ and $j^{\tau }{,}_{\tau }=0$ as expected.

*ii. Poynting vector.* The question we now ask is whether GEM plays a role in radiation problems. Using ${\tilde{F}}_{\mu \nu }$, we can construct, for instance, a “Poynting” vector. Assuming, for simplicity, that $j_{\mu }=0$ in (19), using known vector identities, by integrating over a finite volume we obtain from (19) and (20) the conservation equation

(67) $$\frac{1}{c}\frac{\partial }{\partial t}\int \left({\tilde{E}}^{2}+{\tilde{H}}^{2}\right)dV=-2\oint \vec{\tilde{S}}\cdot d\vec{\Sigma }\;,$$

where Σ is the surface bounding *V* and $\vec{\tilde{S}}=\vec{\tilde{E}}\times \vec{\tilde{H}}$ is the gravitational Poynting vector. Both sides of (67) acquire, in fact, the dimensions of an energy flux after multiplication by $G/c^{3}$. We can now calculate the flux of $\vec{\bar{S}}$ at the particle assuming that the momentum of the free particle is $k\equiv k^{3}$ and that the source in *V* emits a plane gravitational wave in the *x*-direction. In this case the wave is determined by the components $\gamma_{22}=-\gamma_{33}$ and $\gamma_{23}$, and we find ${\tilde{E}}_{1}=0$, ${\tilde{E}}_{2}=2R_{0203}J^{03}+2R_{0231}J^{31}$, ${\tilde{E}}_{3}=2R_{0303}J^{03}+2R_{0331}J^{31}$, ${\tilde{H}}_{1}=0$, ${\tilde{H}}_{2}=-4R_{3103}J^{03}-4R_{3113}J^{13}$ and ${\tilde{H}}_{3}=4R_{2103}J^{03}+4R_{2113}J^{13}$. It also follows that $R_{0203}=R_{0231}=R_{2103}-R_{2113}=-{\ddot{\gamma }}_{23}/2$ and $R_{0303}=R_{0331}=R_{3103}=R_{3113}={\ddot{\gamma }}_{22}/2$. The action of $\tilde{S}$ on the quantum particle is directed along the axis of propagation of the wave and results in a combination of oscillations and rotations about the point $x^{\alpha }$ with angular momentum given by $2J^{03}=\left(x^{0}-z^{0}\right)k-k^{0}\left(x^{3}-z^{3}\right)$, $2J^{13}=\left(x^{1}-z^{1}\right)k$ and $2J^{23}=\left(x^{2}-z^{2}\right)k$. A similar motion also occurs in the case of Zitterbewegung [22]. Reverting to normal units, the energy flux associated with this process is

(68) $$\Phi =\left(\omega^{4}G/c^{3}\right)\left\{{\left(\gamma^{23}\right)}^{2}\left[{\left(J^{03}\right)}^{2}+J^{31}J^{03}\right]+{\left(\gamma^{22}\right)}^{2}\left[{\left(J^{03}\right)}^{2}-J^{31}J^{03}-{\left(J^{31}\right)}^{2}\right]\right\}$$

and increases rapidly with the wave frequency ω and the particle’s angular momentum.

*iii. Electromagnetic radiation.* Let us assume that a spinless particle has a charge *q*. Acceleration, whatever its cause, makes the particle radiate electromagnetic waves. The four-momentum radiated away by the particle, while passing through the driving gravitational field ${\tilde{F}}_{\mu \nu }$, is given by the formula

(69) $$\Delta p^{\alpha }=-\frac{2q^{2}}{3c}\int \frac{du_{\beta }}{ds}\frac{du^{\beta }}{ds}dx^{\alpha }=-\frac{2q^{2}}{3c}\int \left({\tilde{F}}_{\mu \nu }u^{\nu }\right)\left({\tilde{F}}^{\mu \delta }u_{\delta }\right)dx^{\alpha }\;,$$

which can be easily expressed in terms of the external fields (6) using the equation of motion of the charge in the accelerating field [23]. At this level of approximation the particle can distinguish uniform acceleration, which gives $\Delta p^{\alpha }∼\int g^{2}dx^{\alpha }$, where *g* is a constant, from a non-local gravitational field, and it radiates accordingly. This is explained by the presence of $R_{\mu \nu \alpha \beta }$ in (6) and is a direct consequence of our use of the equation of geodesic deviation in (69).

When the accelerating field is the wave discussed above, the incoming gravitational wave and the emitted electromagnetic wave have the same frequency ω and the efficiency of the gravity induced production of photons increases as $\omega^{4}k^{2}$.

*iv. Flux quantization.* Flux quantization is the typical manifestation of processes in which the wave function is non-integrable. Of interest is here the presence of the free particle momentum $k^{\alpha }$ in $K_{\lambda }$.

Let us consider for simplicity the case of a rotating superfluid. Then $\gamma_{01}=-\Omega z_{2}/c$, $\gamma_{02}=\Omega z_{1}/c$ and the remaining metric components vanish. The angular velocity Ω is assumed to be constant in time and $k_{3}=0$. Without loss of generality, we can also choose the reference point $x^{\mu }=0$. We find $K_{0}=K_{3}=0$ and

(70) $$K_{1}=-\frac{1}{2}\left[\gamma_{01,2}z^{2}-\gamma_{01}\right]k^{0}-\frac{1}{2}\left[-\gamma_{01,2}z^{0}\right]k^{2}$$

$$K_{2}=-\frac{1}{2}\left[\gamma_{02,1}z^{1}-\gamma_{02}\right]k^{0}-\frac{1}{2}\left[-\gamma_{02,1}z^{0}\right]k^{1}\;.$$

By integrating over a loop of superfluid, the condition that the superfluid wave function be single-valued gives the quantization condition

(71) $$\oint dz^{\lambda }K_{\lambda }=-\frac{\Omega z^{0}}{2c}\oint \left(k^{2}dz^{1}-k^{1}dz^{2}\right)=\frac{\pi \Omega z^{0}}{c}k\varrho =2\pi n\;,$$

where *n* is an integer, $k=\sqrt{k_{1}^{2}+k_{2}^{2}}$ and $\varrho =\sqrt{z_{1}^{2}+z_{2}^{2}}$. The time integrating factor $z^{0}$, extended to *N* loops, becomes $z^{0}=2\pi \varrho \epsilon N/pc$, where $\epsilon^{2}={\left(pc\right)}^{2}+(mc^{{2)}^{2}}$ and p=ℏk. The superfluid quantum of circulation satisfies the condition

(72) $$\Omega \left(\pi \varrho^{2}\right)\epsilon N/c^{2}=n\hbar \;.$$

If the superfluid is charged, then the wave function is single-valued if the total phase satisfies the relation

(73) $$\oint dz^{\lambda }K_{\lambda }+\frac{q}{c}\oint dz^{\lambda }A_{\lambda }=2\pi n\hbar \;,$$

which, for n=0 and zero external magnetic field, leads to $\int \vec{H}\cdot d\vec{\Sigma }=-2\pi^{2}\Omega \varrho^{2}\epsilon N/qc$. In this case, therefore, rotation generates a magnetic flux through Σ and, obviously, a current in the *N* superconducting loops. No fundamental difference is noticed from DeWitt’s original treatment of the problem [24,25,26,27].

## 5. Vortices

The vector $K_{\lambda }$ is non-vanishing only on surfaces ${\tilde{F}}_{\mu \nu }$ that satisfy (19) and (20) and represent the vortical structures generated by $\Phi_{g}$. At a point $z_{\alpha }$ along the path

(74) $$\frac{\partial \Phi_{g}\left(z\right)}{\partial z^{\sigma }}=-\frac{1}{2}\left[\left(\gamma_{\alpha \sigma ,\beta }\left(z\right)-\gamma_{\beta \sigma ,\alpha }\left(z\right)\right)\left(x^{\alpha }-z^{\alpha }\right)-\gamma_{\beta \sigma }\left(z\right)\right]k^{\beta }=K_{\sigma }\left(z,x\right)\;,$$

and

(75) $$\frac{\partial^{2}\Phi_{g}\left(z\right)}{\partial z^{\tau }\partial z^{\sigma }}-\frac{\partial^{2}\Phi_{g}\left(z\right)}{\partial z^{\sigma }\partial z^{\tau }}=R_{\alpha \beta \sigma \tau }\left(x^{\alpha }-z^{\alpha }\right)k^{\beta }\equiv \left[\partial z_{\tau },\partial z_{\sigma }\right]\Phi_{g}\left(z\right)={\tilde{F}}_{\tau \sigma }\left(z,x\right)\;.$$

There may then be closed paths embracing the singularities along which the particle wave function must be made single-valued by means of appropriate quantization conditions [28]. It also follows from (75) that ${\tilde{F}}_{\mu \nu }$ is a vortex along which the scalar particles are dragged with acceleration

(76) $$\frac{d^{2}z_{\mu }}{ds^{2}}=u^{\nu }\left(u_{\mu ,\nu }-u_{\nu ,\mu }-R_{\mu \nu \alpha \beta }\left(x^{\alpha }-z^{\alpha }\right)u^{\beta }\right)\;,$$

and relative acceleration

(77) $$\frac{d^{2}\left(x_{\mu }-z_{\mu }\right)}{ds^{2}}={\tilde{F}}_{\mu \lambda }u^{\lambda }=R_{\mu \beta \lambda \alpha }\left(x^{\alpha }-z^{\alpha }\right)u^{\beta }u^{\lambda }\;,$$

in agreement with the equation of geodesic deviation [28]. Notice that in (76) the vorticity is entirely due to $R_{\mu \nu \alpha \beta }J^{\alpha \beta }$ and that $\frac{d^{2}z_{\mu }}{ds^{2}}=0$ when the motion is irrotational. This also applies when $R_{\mu \nu \alpha \beta }=0$, in which case the vortices do not develop. Similarly, vortices do not form if $k^{\alpha }=0$. Each gravitational field produces a distinct vortex whose equations are (19) and (20); the vortex dynamics are given by (76) and (77); and the topology of the object is supplied by $\Phi_{G}$. Though we started from a quantum wave equation, the vortices generated are purely classical because $\gamma_{\mu \nu },K_{\lambda }$ and ${\tilde{F}}_{\alpha \beta }$ are classical and the particles interact with gravity as classical particles do. In addition, ϕ and $\phi_{0}$ coexist with the vortices generated by $\Phi_{g}$ in the ground state. The field ${\tilde{F}}_{\mu \nu }$ emerges as a property of gravitation when this interacts with particles described by quantum wave equations. Its range is that of $\gamma_{\mu \nu }$. ${\tilde{F}}_{\alpha \beta }$ vanishes on the line $x^{\alpha }-z^{\alpha }=0$ along which $K_{\lambda }$ can also be eliminated by a gauge transformation. In this case we can say that the line is entirely occupied by $\phi_{0}$. Obviously $\Phi_{G}=0$ on the nodal lines of ϕ where it loses its meaning. Notice that the right hand side of (16) can also be replaced by its dual. This is equivalent to interchanging the “magnetic” with the “electric” components of $R_{\mu \nu \alpha \beta }$ and the corresponding vortex types.

We finally stress that it is the transformation (12) that induces the macroscopic phenomena governed by the classical Equations (19) and (20). The same transformation thus provides a mechanism by which a classical theory of gravity can be connected with quantum theory.

## 6. A Minimal Lagrangian

The coordinate *x* that refers to the total displacement along the path in the local coordinate system has no role in what follows and can be dispensed with.

The simplest possible Lagrangian in which the features discussed in the previous sections can be accommodated is [29]

(78) $$L=-\frac{1}{4}{\tilde{F}}_{\alpha \beta }{\tilde{F}}^{\alpha \beta }+{\left[\left(\partial_{\mu }-iK_{\mu }\right)\phi \right]}^{*}\left[\left(\partial^{\mu }+iK^{\mu }\right)\phi \right]-\mu^{2}\phi^{*}\phi \;,$$

where $\mu^{2}<0$. The second term of L contains the first order gravitational interaction $\gamma_{\mu \nu }{\left[\left(\partial^{\mu }-iK^{\mu }\right)\phi \right]}^{*}\left[\left(\partial^{\nu }+iK^{\nu }\right)\phi \right]∼-\gamma_{\mu \nu }\partial^{\mu }\partial^{\nu }\phi_{0}$ met above. By varying L with respect to $\phi^{*}$ and by applying a gauge transformation to $K_{\alpha }$, we find, to $O(\gamma_{\mu \nu })$,

(79) $$\left[\partial^{2}+m^{2}+\gamma_{\mu \nu }\partial^{\mu }\partial^{\nu }\right]\phi \left(z\right)≃0\;,$$

and $-\mu^{2}$ has now been changed into $m^{2}>0$ because the Goldstone boson has disappeared; the remaining boson is real and so must be its mass [29]. Equation (79) is identical to (9) and its solution is still represented by the boson transformation (12). However, a variation of L with respect to $K_{\alpha }$ now gives

(80) $$\partial_{\nu }{\tilde{F}}^{\mu \nu }={\tilde{J}}^{\mu }=i\left[\left(\phi^{*}\partial^{\mu }\phi \right)-\left(\partial^{\mu }\phi^{*}\right)\phi \right]-2K^{\mu }\phi^{*}\phi \;$$

from which, on using (12) and a gauge transformation, we obtain the field equation

(81) $$\partial^{2}K_{\mu }+2K_{\mu }\phi^{2}=0\;,$$

that shows that $K_{\mu }$ has acquired a mass. By expanding $\phi =v+\rho \left(z\right)/\sqrt{2}$, we find that the mass of $K_{\mu }$ is *v* and its range $∼v^{-1}$. Any metrical theory of gravity selected remains valid at distances greater than $v^{-1}$, but not so near or below $v^{-1}$. The screening current in (81) determines a situation analogous to that of vortices of normal electrons inside type-II superconductors where the electron normal phase is surrounded by the condensed, superconducting phase. The fundamental difference from the approach followed in the first two sections is represented by (81) that now becomes a constraint on $K_{\lambda }$. It can be satisfied by requiring that $\left(\partial^{2}+v^{2}\right)\gamma_{\alpha \beta }=0$. No other changes are necessary. On the other hand this condition can be applied directly in Section 1 and Section 2 without making use of L. ${\tilde{F}}_{\mu \nu }$ again vanishes when $z^{\alpha }-x^{\alpha }=0$, which indicates that the line $z^{\alpha }-x^{\alpha }=0$ can only be occupied by the normal phase. As before, the field ${\tilde{F}}_{\mu \nu }$ is classical and emerges as a property of gravitation when it interacts with quantum matter. The range of interaction can obviously be very short if *v* is large.

## 7. The One-Dimensional Model

A lattice gas model can be used to calculate the alignment per particle and correlation length that follow from gravity induced condensation. The properties of a many-particle system satisfying (12) follow from $H=g_{\mu \nu }P^{\mu }P^{\nu }≃m^{2}+2\gamma_{\mu \nu }k^{\mu }k^{\nu }$ which strongly resembles the energy function of the Ising model. A difference is represented here by the vectors $k_{\alpha }$ (or $P_{\alpha }$) that replace the Ising spin variables $\sigma_{i}$, which are numbers that can take the values ±1. It is, however, known that a lattice gas model [30], equivalent to the Ising model, can be set up in which the particles are restricted to lie only on the *N* sites of a fine lattice, instead of being allowed to occupy any position in space-time. Then one can associate with each site *i* a variable $s_{i}=\left(1+\sigma_{i}\right)/2$ which takes the value 1 if the site is occupied by a vector $k_{\alpha }$ and the value 0 if it is empty. Any distribution of the particles can be indicated by the set of their site occupation numbers $s_{1},\ldots .s_{N}$. By replacing $k^{\mu }k^{\nu }$ in *H* with their average $k^{2}\eta^{\mu \nu }/4$ over the angles in Minkowski space and restricting the interaction to couples of nearest sites, one obtains

(82) $$H=-\frac{m}{4}\gamma \left(\sum_{k=1}^{N}s_{k}s_{k+1}\right)\;,$$

where $\gamma \equiv \gamma_{\mu \nu }\eta^{\mu \nu }$. By imposing periodic conditions $s_{N+1}=s_{1}$ along the hypercylinder with axis parallel to the time-axis, the partition function becomes

(83) $$Z=\sum_{s_{1}}\ldots \sum_{s_{N}}\exp\left(\beta \epsilon \sum_{k=1}^{N}s_{k}s_{k+1}\right)\;,$$

where β≡1/T and ϵ≡mγ/4 contains the gravitational contribution due to $\gamma_{\mu \nu }$. This one-dimensional Ising model can be solved exactly [30]. Equation (83) can be rewritten as $Z=\sum_{s_{1}}<s_{1}\left|{\tilde{M}}^{N}\right|s_{1}>=Tr\left({\tilde{M}}^{N}\right)=\lambda_{+}^{N}+\lambda_{-}^{N}$ and the eigenvalues of $\tilde{M}$ are $\lambda_{+}=2\cosh\left(\beta \epsilon \right)$ and $\lambda_{-}=2\sinh\left(\beta \epsilon \right)$. As N→∞, $\lambda_{+}$ makes a larger contribution than $\lambda_{-}$, $N^{-1}\ln Z\to \ln\left(\lambda_{+}\right)$ and the Helmholtz free energy per site is $F/N=-{\left(N\beta \right)}^{-1}\ln Z\to -\beta^{-1}\ln\left(\lambda_{+}\right)$. The alignment per particle for large values of *N* is

(84) $$\Gamma \equiv -\frac{1}{N}\frac{\partial F}{\partial \epsilon }∼\frac{1}{\beta }\frac{d\ln\lambda_{+}}{d\epsilon }=\tanh\left(\beta \epsilon \right)\;,$$

which yields the gravitational correction contained in ϵ. It also follows from (84) that there is no spontaneous momentum alignment (Γ=0 when ϵ=0) and that complete alignment Γ=1 is possible only for T→0. In fact F→−Nϵ in the limit T→0 for completely aligned momenta and one can say that there is a phase transition at T=0, but none for T>0. It follows from (84) that the value of Γ depends on the gravitational contribution γ. It also follows that there is no alignment (Γ=0) for T→∞ (for any γ and *m*), or for γ=0 (no gravity and any *T*). According to (84), complete alignment Γ=1 can be achieved only at T=0, which, as shown below, is not, however, a critical temperature in the model.

The correlation length per unit of lattice spacing is

(85) $$\xi ∼\frac{1}{2}\exp\left(2\beta \epsilon \right)\;,$$

which gives ξ=∞ at T=0 and ξ=1/2 at T=∞ where thermal agitation can effectively disjoin neighboring sites.

One can also define a gravitational susceptibility as dΓ/dγ. One finds

(86) $$\frac{d\Gamma }{d\gamma }=\frac{m\beta }{4\cosh^{2}\left(\beta \epsilon \right)}\geq 0\;$$

always. It follows that when βϵ≪1, tanh(βϵ)≈βϵ=γm/4T, and

(87) $$\frac{d\Gamma }{d\gamma }≃\frac{m}{4T}=2.9\times 10^{12}\frac{m(GeV)}{T(K)}\;,$$

which is reminiscent of Curie’s law.

Interesting aspects of the problem are revealed by calculating the value of *T* for which $1/\cosh^{2}\left(\beta \epsilon \right)$ has an extremum. The derivative of dΓ/dγ with respect to *T* vanishes when 2ytanh(y)=1 which gives y=mγ/4T=0.77. The temperature at the extremum is

(88) $$T_{m}\left(K\right)=\frac{m\gamma }{4\times 0.77}=\frac{m(GeV)\gamma }{2.65\times 10^{-13}}\;,$$

and the corresponding value of the susceptibility is

(89) $${\left(\frac{d\Gamma }{d\gamma }\right)}_{m}=\frac{m}{4T_{m}\cosh^{2}\left(\frac{m\gamma }{4T_{m}}\right)}≃\frac{0.45}{\gamma }\;,$$

which is independent of *m*. All materials therefore respond to changes in gravity in a universal way that depends only on the source, rather than on the material itself. Table 1 lists the values of ${\left(d\Gamma /d\gamma \right)}_{m}$ for some relevant astrophysical objects. In $T_{m}$, the nucleon mass m∼0.9 GeV has been used for simplicity. In general, even a small value of γ is sufficient to saturate the alignment of momenta over a range of temperatures, which is narrow because of the sharpness of dΓ/dγ. At $T=T_{m}$, the correlation length is only ξ≃2.33 which is rather small. This is expected because the values T=0 and $T≃T_{m}$ correspond respectively to states of low and high thermal agitation in which the system changes from a high to a small correlation. It is therefore necessary to consider the value of ξ at a particular temperature.

In all cases where βϵ≪1, one finds T≫1 and (dΓ/dγ)≪1/γ. This is the high *T* case. If βϵ≫1, then T≪(1/4)γm and $0\leq \left(d\Gamma /d\gamma \right)<1.2\times 10^{12}m\left(GeV\right)/T\left(K\right)$. For Earth, T∼300 K yields $d\Gamma /d\gamma ∼1.6\times 10^{7}$ and $\xi ∼1.8\times 10^{7}$.

**Figure 1:**
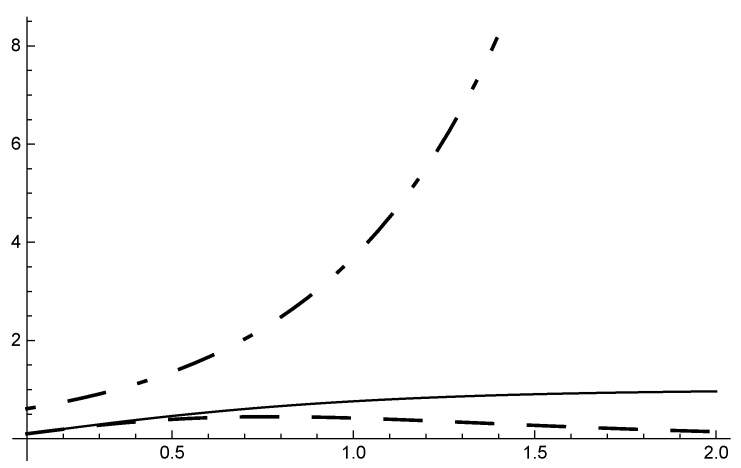
ξ (dot-dashed); Γ (continuous); ∂Γ/∂γ (dashed) for 0<γ<2.

**Figure 2:**
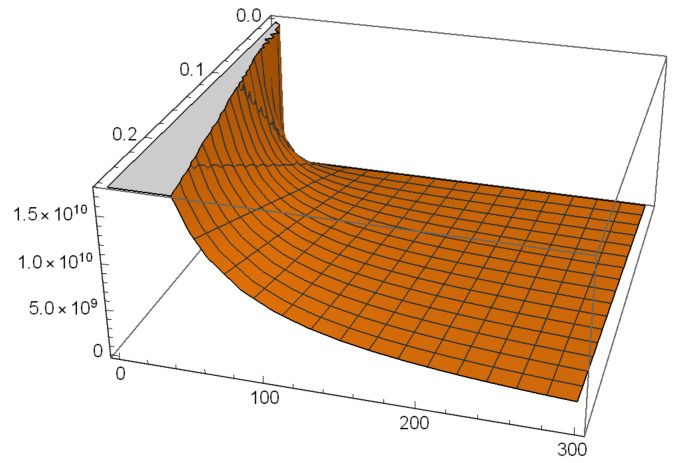
$\gamma \frac{d\Gamma }{d\gamma }$ for 0≤T≤300 K and 0≤γ≤0.26.

It follows from Table 1 and from Figure 1 and Figure 2, that condensation effects can be large even though γ is small, provided ξ retains a reasonable value. This is an unusual occurrence in gravitation. The effect should be observable by comparing $\frac{\partial \Gamma }{d\gamma }$ at $T=T_{m}$ for different materials.

The oscillations of $P_{\mu }$ are similar to spin waves and obey the dispersion relation [28,31]

(90) $$\omega =\frac{ms\gamma }{2}\left(1-\cos\ell a\right)\;,$$

where *s* is the spin magnitude at a site, $\ell =|\vec{\ell }|$ is the spin-wave momentum and *a* is the lattice spacing. Upon quantization, spin waves give rise to quasiparticles that, by analogy with magnons, shall be called “gravons”. For oscillations of small amplitude and on using (90), one obtains $\omega ≃ms\gamma {\left(\ell a\right)}^{2}/2=\ell^{2}/2m^{*}$, where $m^{*}$ is taken as the gravon’s effective mass. If the lattice subdivision is very fine so that $a∼m^{-1}$, then $m^{*}=m/s\gamma$ can become large for small γ and the oscillation frequency of these waves is very low. Magnons can, of course, be created in laboratory experiments by scattering neutrons against an ordered magnetic structure. The analogous gravon experiment in Earth or near space laboratories, seems to be precluded by the high values of $m^{*}$ for small γ. It would be useful, for astrophysical purposes, to derive a gravon’s spectrum and distribution. For sufficiently small changes of γ, the energy of a mode of energy $\omega_{\ell }=\ell^{2}/2m^{*}$ and $n_{\ell }$ gravons is that of a harmonic oscillator and the gravon distribution is that of Planck. When $\omega_{\ell }\ll T$, the number of gravons per unit volume is

(91) $$\sum_{\ell }n_{\ell }≃8\left(0.0587\right){\left(\frac{T}{ms\gamma a^{2}}\right)}^{3/2}\;,$$

also reminiscent of Bloch $T^{3/2}$ law for magnetism. As indicated by Figure 2, the approach to the Curie point is less sharp than in magnetism. This is due to the mitigating action that a small γ has on 1/T in ϵ as T→0. Curie’s law and (91) suggest $T_{c}=0$ as a critical temperature. It is shown in the next section that this choice is, however, inappropriate.

Finally, the radiation spectrum of gravons produced by a proton a distance *b* from a star of mass *M* can be calculated from the power radiated in the process of $p\to p^{\prime }+\tilde{\gamma }$ using the expression

(92) $$W=\frac{1}{8{\left(2\pi \right)}^{2}}\int \delta^{4}\left(P-p^{\prime }-\ell \right)\frac{{\left|M\right|}^{2}}{Pp_{0}^{\prime }}d^{3}p^{\prime }d^{3}\ell \;,$$

where

(93) $$\Sigma |M_{p\to p^{\prime }\tilde{\gamma }}{|}^{2}=e^{2}\left[-4\left({p^{\prime }}^{\alpha }\Phi_{G,\alpha }\right)+8\left(p^{\alpha }\Phi_{G,\alpha }\right)\right]\;,$$

and $\tilde{\gamma }$ represents the gravon. The process is similar to that of magnon production by neutron scattering by a magnetic structure. The quantum mechanical power spectrum of $\tilde{\gamma }$ is

(94) $$\frac{dW}{d\ell }≃\frac{e^{2}\ell }{\pi }\left(\frac{GM}{b}\right)\;,$$

where $p=|\vec{p}|>m_{p}$ is the momentum of the incoming nucleon and pGM/b<ℓ≤p to satisfy the requirement |cosϑ|≤1, where ϑ is the emission angle. Results (90) and (94) agree for ℓa≪1.

If $\omega_{\ell }\ll T$, the total number of gravons can be written in the form

(95) $$\tilde{N}≃\frac{4T}{ms\gamma a}∼\frac{4TL}{ms\gamma a^{2}\tilde{n}}\;,$$

where $\tilde{n}=N/V$ and $L∼\tilde{n}a$ is the typical size of the system. From $\tilde{N}/N\leq 1$, one gets $T\leq ms\gamma \tilde{n}a^{2}/4L$ which can be satisfied for sufficiently large *L*.

## 8. A Two-Dimensional Model

Not all ordered phases can exist, because the number of space dimensions plays a role in phase changes. Changes to ordered phases can survive only if they are stable against long wavelength fluctuations. Consider, for instance, a system of particles that is invariant under translations in a space *V* of *d* dimensions. Representing the deviation of the particles from the equilibrium position by $q\left(x\right)=\left(1/V\right)\sum_{k,r}e^{ik\cdot x}q_{i}\left(k\right)$, where *i* indicates the normal modes and *k* extends up to some value, the normal modes energy is $T=\omega_{i}^{2}\left(k\right){\left|q_{i}\left(k\right)\right|}^{2}$ by the theorem of equipartition of energy. Then for V→∞, $<q{\left(x\right)}^{2}>=\Sigma_{i}\int d^{d}k2T/\omega_{i}^{2}$. If the continuous symmetry has been broken spontaneously, the excitations whose frequencies $\omega_{i}\left(k\right)∼k$, vanish as k→0 and give the low frequency limit <*q*${}^{2}$> $∼\Sigma_{i}\int dkk^{d-1}/k^{2}$ which diverges for d≤2. Hence the lowest critical dimension is d=2, below which order is destroyed by long wavelength fluctuations.

Consider, therefore, the case *d* = 2. The quickest way to obtain the relevant expressions for a two-dimensional model is to restart from (82) and replace the vectors $s_{i}$ with classical vectors constrained to lie in the $s_{x}s_{y}$–plane. Then $s_{i}=\left(s_{ix},s_{iy}\right)$ and $s_{ix}^{2}+s_{iy}^{2}=1$ and

(96) $$H=-\frac{m\gamma s^{2}}{4}\Sigma_{i,j}\cos\left(\theta_{i}-\theta_{j}\right)$$

if *i* and *j* are neighbors; H=0 otherwise. If the neighboring angles are close in value, then by neglecting an irrelevant constant, $H≃-\left(m\gamma s^{2}/8\right)\Sigma_{\vec{R}}\Sigma_{\vec{a}}{\left[-\theta \left(\vec{r}\right)+\theta \left(\vec{r}+\vec{a}\right)\right]}^{2}$, where $\vec{r}+\vec{a}$ is the nearest neighbor of $\vec{r}$. When replacing the finite differences with derivatives one gets

(97) $$H=\frac{1}{8}m\gamma s^{2}\int d^{2}r\vec{\nabla }\theta \left(\vec{r}\right)\cdot \vec{\nabla }\theta \left(\vec{r}\right)\;.$$

These excitations are vortices for which $\vec{\nabla }\theta =n\vec{a_{\theta }}/r$ and *n* is an integer. The energy of an isolated vortex is $E=\left(1/8\right)m\gamma s^{2}\int_{a}^{L}dr/r=\left(1/8\right)m\gamma s^{2}\ln\left(L/a\right)$, where *L* is the linear dimension of the vortex. The entropy associated with a single vortex is $S=\ln{\left(L/a\right)}^{2}$ and the change in free energy due to the formation of a vortex is

(98) $$\Delta G=\left(\frac{1}{8}m\gamma s^{2}n^{2}-2T\right)\ln\frac{L}{a}\;,$$

which is positive for

(99) $$T<\frac{1}{8}m\gamma s^{2}n^{2}\equiv T_{c}\;.$$

Isolated vortices do not therefore form for $T\leq T_{c}$. At low temperatures the state of the system consists of an equilibrium density of bound vortices. At $T>T_{c}$ the vortices become unbound and the condensed phase is destroyed.

The condition $\tilde{N}/N\leq 1$ gives in two dimensions

(100) $$\tilde{N}≃\frac{4T}{\tilde{n}ms\gamma a^{2}}\ln\left(\frac{L}{a}\right)\leq 1\;,$$

which can be satisfied for $0\leq T\leq m\tilde{n}s\gamma a^{2}/4\ln\left(L/a\right)$ provided L/a≥1.

Given the important role of space dimensionality in critical phenomena, one may wonder about the behavior of $\tilde{N}$ in dimensions higher than two. In three dimensions the condition $\tilde{N}/N≃2T/\left(\pi \tilde{n}ms\gamma a^{3}\right)\leq 1$ can be satisfied at all *T* for sufficiently high values of $\tilde{n}∼L/a$.

## 9. Summary and Conclusions

A rotationally-invariant quantum system acquires a privileged direction in the course of its evolution in curved space-time. This symmetry breaking takes the form of a space-time-dependent ground state (12) and of a topological singularity in (13) that leads to the phenomenon of condensation in an ensemble of like particles. In the re-distribution of degrees of freedom that follows symmetry breaking, the gravitational field is represented by the two-point potential $K_{\mu }\left(z,x\right)$. The tensor ${\tilde{F}}_{\mu \nu }=K_{\nu ,\mu }-K_{\mu ,\nu }$ satisfies Maxwell-type equations and depends on the metric tensor. The potential $K_{\lambda }$ suggests the introduction of the notion of the quasiparticle, because gravity affects, in general, the dispersion relations of the particles with which it interacts, as shown by (29), and because it carries with itself information about matter through $k_{\alpha }$.

A simple Lagrangian was introduced to illustrate how $K_{\mu }\left(x\right)$ acquires a mass *v* and a range $v^{-1}$. Above $v^{-1}$ the gravitational interaction is conveyed by $\gamma_{\mu \nu }$, but below this range, when interacting with matter, the gravitational action is carried by $K_{\lambda }$. The screening current associated with a massive $K_{\lambda }$ resembles what occurs with vortices of normal electrons in type-II superconductors where the electron normal phase is surrounded by the condensed superconducting phase. The normal phase $\phi_{0}$ remains shielded from the external gravitational field, a situation that is of interest in dark matter studies. The field ${\tilde{F}}_{\mu \nu }$ is classical and emerges as a property of gravitation when the latter interacts with quantum matter.

Some applications of GEM have been examined in some detail.

The equations obeyed by ${\tilde{F}}_{\mu \nu }$ do not contemplate the presence of “gravitomagnetic” charges. This follows immediately from (20) and (4).

The “gravitoelectric” charge density follows from $j^{\tau }=-8\pi G/c^{4})\left(T^{\beta \tau }k_{\beta }\right)$ when τ=0, and after integration over space, can be interpreted as the amount of work done by the gravitational field to deflect the particle by an amount $x_{\alpha }-z_{\alpha }$ in a time dt.

Some particular aspects of the behavior of $K_{\lambda }$ have been examined. We have found that when $j_{\mu }=0$, scale invariance assures that a gas of gravitons satisfies Planck’s radiation law, but that this is no longer so, in principle, for non-pure gravitational fields.

$K_{\lambda }$ also determines the equations of motion of a particle through (30)–(32) and (21). We have found that the motion follows a geodesic only if the quantum particle chooses, among all available paths, that for which $Dk_{\alpha }/Ds=0$. Along this particular path the principle of equivalence is satisfied. We have then shown that the particle motion is contained in the solution (12) of the covariant KG equation.

We have also studied quantum mechanical tunneling through a horizon and derived a covariant and canonical invariant expression for the transition amplitude. Though the approximation looks ill-suited to deal with regions of space-time close to a gravitational horizon, the approximation reproduces the Unruh temperature exactly in the case of the Rindler metric. No corrections of and no effects due to $k_{\mu }$ have been found for the standard result of $O(\gamma_{\mu \nu })$. Higher order approximations can be calculated by applying (60).

As ${\tilde{F}}_{\mu \nu }$ satisfies Maxwell-like equations, it is also possible to define a Poynting vector and a flux of energy and angular momentum at the particle so that the particle’s motion can be understood as a sequence of oscillations and rotations similar to what is found in the case of Zitterbewegung [22].

Use of $K_{\lambda }$ in problems where gravity accelerates a charged particle and electromagnetic radiation is produced offers a rather immediate relationship between the loss of energy-momentum by the quantum particle and the driving gravitational field. These processes could give sizable contributions for extremely high values of ω. Astrophysical processes like photoproduction [32] and synchrotron radiation [33] have been discussed in the literature and are worthy of re-consideration in view of the present results. An advantage from the point of view of detecting high frequency gravitational radiation, for which detection schemes are in general difficult to conceive, is represented by the efficiency of the graviton–photon conversion rate and by the high coupling afforded by a radio receiver over, for instance, a mechanical one. This would enable, in principle, a spectroscopic analysis of the signal.

In the last problem considered, we have calculated the flux of $K_{\lambda }$ in the typical quantum case of a non-integrable wave function. Here too, it is possible to isolate quantities of physical interest, such as magnetic flux or circulation, despite the non-intuitive character of $\oint dz^{\lambda }K_{\lambda }$. Unlike [24], our procedure and results are fully relativistic. They can be applied directly to boson condensates in boson stars [34].

Covariant wave equations have solutions (12) and (21) that are space-time-dependent transformations of the vacuum. The resulting degeneracy of the ground states produces Nambu-Goldstone excitations which break the rotational symmetry of the system. The quasiparticles generated, or gravons, are oscillations that obey the dispersion relation (90) and have an effective mass.

In the one-dimensional Ising model considered, the order parameter is the generalized momentum $\Phi_{G,\mu }$, along which the particle momenta tend to align. The motion of the particles in the direction of $\Phi_{G,\mu }$ is along geodesics. Off them, along the variable *z*, $\Phi_{g}$ is topologically singular and length scales change. The phase singularities of $\Phi_{g}$ give rise to quantized vortices [35], and the particle motion along the hypersurfaces ${\tilde{F}}_{\alpha \beta }\neq 0$ satisfies the equation of deviation. Phase singularities give rise to strings of silence in acoustics; lines of magnetic flux in magnetism; and vortices in optics, in superfluids and superconductors [35]. The multivalued nature of $\Phi_{g}$ leads to the loss of a standard of length in the region of critical phenomena. For space-time loops linking the regions of singularity, one must have

(101) $$\oint_{\Gamma }dz^{\lambda }K_{\lambda }\left(z\right)=\oint_{\Gamma }dz^{\lambda }\frac{\partial \Phi_{g}}{\partial z_{\lambda }}=\int_{\Sigma }d\sigma^{\lambda \mu }{\tilde{F}}_{\lambda \mu }=2n\pi \;,$$

where Σ is a surface bound by Γ. Outside the critical region the change in length still vanishes around paths Γ that do not link any singularity. The field ${\tilde{F}}_{\sigma \tau }$ vanishes on the line $x_{\sigma }-z_{\sigma }=0$ which is entirely occupied by the normal phase. In addition, the choice $\partial^{2}\gamma_{\alpha \beta }\neq 0$ in (18), would give a Meissner-type effect for ${\tilde{F}}_{\sigma \tau }$. The result is here analogous to that of superconducting strings. It is also remarkable that a singularity in a quantum mechanical wave function produces a field ${\tilde{F}}_{\sigma \tau }$ that is entirely classical.

The effect of gravity on some parameters that characterize the critical behavior of a quantum system of scalar particles can be calculated. For instance, the gravitational susceptibility dΓ/dγ is always positive and obeys a Curie-type law. The susceptibility can be understood as a measure of the reaction of the system to a gravitational field and is analogous to the magnetic susceptibility. There are, of course, no gravitational dipoles in an ensemble of quantum particles because Einstein’s gravity is always attractive. There are only the particle momentum vectors in the lattice gas model considered. Therefore, dΓ/dγ indicates only how the momentum alignment per particle changes when the gravitational parameter γ changes. Since dΓ/dγ always satisfies (86), the response to changes in γ can be termed “paragravitational”. The increase is larger as T→0 because at lower temperatures thermal agitation subsides and correlation is preserved.

The number of quantized spin-waves, or gravons, per unit volume, follows a $T^{3/2}$ law; the emission cross-section is low; and the gravon spectrum depends linearly on the momentum *ℓ*, both classically and quantum mechanically, if ℓa≪1.

In the one-dimensional case one cannot say that T=0 is the critical temperature of the model. The lowest critical dimension is 2 and the simplest two-dimensional system is the XY-model. A critical temperature is in fact given by (99), below which isolated vortices do not exist; only bound vortices do. Symmetry breaking has a topological origin also in the XY-model. Due to the important developments that have recently taken place in the field of condensed matter physics, such as block spin models and renormalization, the prospects of extending the results to dimensions greater than one look promising and are under consideration.

Summarizing, the solution (12) and its extension (21) introduce topological singularities and induce condensation. This gives rise to the classical and quantum aspects of GEM discussed above when gravity interacts with quantum matter. 

## Figures and Tables

**Table 1:** Maximum values of dΓ/dγ for some astrophysical objects.

	γ	$T_{m}$	${\left(d\Gamma /d\gamma \right)}_{m}$
Earth	$10^{-9}$	3391	$4.5\times 10^{8}$
Sun	$2\times 10^{-6}$	$7.2\times 10^{6}$	$2.3\times 10^{5}$
Neutron star	0.26	$8.8\times 10^{11}$	1.73
White dwarf	$10^{-3}$	$3.4\times 10^{9}$	450

## References

[1] DeWitt B.S., DeWitt C., DeWitt B. (1964). Dynamical Theory of Groups and Fields. Relativity, Groups and Topology, Les Houches Lectures 1963.

[2] Lambiase G., Papini G., Punzi R., Scarpetta G. (2005). Neutrino optics and oscillations in gravitational fields. Phys. Rev. D.

[3] Cai Y.Q., Papini G. (1991). Neutrino helicity flip from gravity-spin coupling. Phys. Rev. Lett..

[4] Papini G., Rizzi G., Ruggiero M.L. (2004). Relativity in Rotating Frames.

[5] Papini G. (2008). Spin–rgravity coupling and gravity-induced quantum phases. Gen. Relativ. Gravit..

[6] Ruse H.S. (1931). Taylor’s theorem in the tensor calculus. Proc. Lond. Math. Soc..

[7] Synge J.L. (1966). Relativity: The General Theory.

[8] Papini G. (2007). Spin-2 particles in gravitational fields. Phys. Rev. D.

[9] Papini G., Scarpetta G., Feoli A., Lambiase G. (2009). Optics of spin-1 particles from gravity-induced phases. Int. J. Mod. Phys. D.

[10] Feynman R.P., Hibbs A.R. (1965). Quantum Mechanics and Path Integrals.

[11] Lanczos C. (1970). The Variational Principles of Mechanics.

[12] Skrotskii G.V. (1957). On the influence of gravity on the light propagation. Dokl. Akad. Nauk SSSR.

[13] Kopeikin S., Mashhoon B. (2002). Gravitomagnetic effects in the propagation of electromagnetic waves in variable gravitational fields of arbitrary-moving and spinning bodies. Phys. Rev. D.

[14] Ballentine L.E. (1990). Quantum Mechanics.

[15] Akhmedov E.T., Akhmedova V., Singleton D. (2006). Hawking temperature in the tunneling picture. Phys. Lett. B.

[16] Takagi S. (1986). Vacuum noise and stress induced by uniform acceleration hawking-unruh effect in rindler manifold of arbitrary dimension. Prog. Theor. Phys. Suppl..

[17] De Gill A., Singleton D., Akhmedova V., Pilling T. (2010). A WKB-like approach to Unruh radiation. Am. J. Phys..

[18] Unruh W.G. (1976). Notes on black-hole evaporation. Phys. Rev. D.

[19] Crispino L.C., Higuchi A., Matsas G.E. (2008). The Unruh effect and its applications. Rev. Mod. Phys..

[20] Benedetto E., Feoli A. (2015). Unruh temperature with maximal acceleration. Mod. Phys. Lett. A.

[21] Misner C.W. (1969). Absolute zero of time. Phys. Rev..

[22] Papini G. (2012). Zitterbewegung and gravitational Berry phase. Phys. Lett. A.

[23] Papini G. (2014). Covariance and gauge invariance in relativistic theories of gravity. Mod. Phys. Lett. A.

[24] DeWitt B.S. (1966). Superconductors and gravitational drag. Phys. Rev. Lett..

[25] Papini G. (1966). London moment of rotating superconductors and Lense-Thirring fields of general relativity. Nuovo C..

[26] Bakke K., Furtado C., Nascimento J.R. (2011). Geometric quantum phase in the spacetime of topological defects. J. Phys. Conf. Ser..

[27] Castro L.B. (2016). Noninertial effects on the quantum dynamics of scalar bosons. Eur. Phys. J. C.

[28] Papini G. (2017). Classical and quantum aspects of particle propagation in external gravitational fields. Int. J. Mod. Phys. D.

[29] Itzykson C., Zuber J.-B. (1980). Quantum Field Theory.

[30] Baxter R.J. (1982). Exactly Solved Models in Statistical Mechanics.

[31] Kittel C. (1996). Introduction to Solid State Physics.

[32] Papini G., Valluri S.R. (1977). Gravitons in Minkowski space-time interactions and results of astrophysical interest. Phys. Rep..

[33] Misner C.W. (1972). Interpretation of Gravitational-Wave Observations. Phys. Rev. Lett..

[34] Chavanis P.-H., Harko T. (2012). Bose-Einstein Condensate general relativistic stars. Phys. Rev. D.

[35] Berry M., Balian R. (1981). Singularities in Waves And, Rays.

